# Using optical coherence tomography and intravascular ultrasound imaging to quantify coronary plaque cap thickness and vulnerability: a pilot study

**DOI:** 10.1186/s12938-020-00832-w

**Published:** 2020-11-30

**Authors:** Rui Lv, Akiko Maehara, Mitsuaki Matsumura, Liang Wang, Qingyu Wang, Caining Zhang, Xiaoya Guo, Habib Samady, Don P. Giddens, Jie Zheng, Gary S. Mintz, Dalin Tang

**Affiliations:** 1 School of Biological Science and Medical Engineering, Southeast University, #2 SiPailou, Nanjing, China; 2 The Cardiovascular Research Foundation, Columbia University, New York, USA; 3 School of Science, Nanjing University of Posts and Telecommunications, Nanjing, China; 4 Department of Medicine, Emory University School of Medicine, Atlanta, GA USA; 5 The Wallace H. Coulter Department of Biomedical Engineering, Georgia Institute of Technology, Atlanta, GA USA; 6 Mallinckrodt Institute of Radiology, Washington University, St. Louis, MO USA; 7 Mathematical Sciences Department, Worcester Polytechnic Institute, 100 Institute Road, Worcester, MA 01609 USA

**Keywords:** OCT, IVUS, Fibrous cap, Vulnerable plaque, Cap thickness

## Abstract

**Background:**

Detecting coronary vulnerable plaques in vivo and assessing their vulnerability have been great challenges for clinicians and the research community. Intravascular ultrasound (IVUS) is commonly used in clinical practice for diagnosis and treatment decisions. However, due to IVUS limited resolution (about 150–200 µm), it is not sufficient to detect vulnerable plaques with a threshold cap thickness of 65 µm. Optical Coherence Tomography (OCT) has a resolution of 15–20 µm and can measure fibrous cap thickness more accurately. The aim of this study was to use OCT as the benchmark to obtain patient-specific coronary plaque cap thickness and evaluate the differences between OCT and IVUS fibrous cap quantifications. A cap index with integer values 0–4 was also introduced as a quantitative measure of plaque vulnerability to study plaque vulnerability.

**Methods:**

Data from 10 patients (mean age: 70.4; m: 6; f: 4) with coronary heart disease who underwent IVUS, OCT, and angiography were collected at Cardiovascular Research Foundation (CRF) using approved protocol with informed consent obtained. 348 slices with lipid core and fibrous caps were selected for study. Convolutional Neural Network (CNN)-based and expert-based data segmentation were performed using established methods previously published. Cap thickness data were extracted to quantify differences between IVUS and OCT measurements.

**Results:**

For the 348 slices analyzed, the mean value difference between OCT and IVUS cap thickness measurements was 1.83% (*p *= 0.031). However, mean value of point-to-point differences was 35.76%. Comparing minimum cap thickness for each plaque, the mean value of the 20 plaque IVUS-OCT differences was 44.46%, ranging from 2.36% to 91.15%. For cap index values assigned to the 348 slices, the disagreement between OCT and IVUS assignments was 25%. However, for the OCT cap index = 2 and 3 groups, the disagreement rates were 91% and 80%, respectively. Furthermore, the observation of cap index changes from baseline to follow-up indicated that IVUS results differed from OCT by 80%.

**Conclusions:**

These preliminary results demonstrated that there were significant differences between IVUS and OCT plaque cap thickness measurements. Large-scale patient studies are needed to confirm our findings.

## Background

Cardiovascular disease is the leading cause of death worldwide. Most cardiovascular events such as heart attack and stroke are linked to development and rupture of vulnerable plaques. Plaque morphological features such as thin fibrous cap and large lipid-rich necrotic pools have been recognized as the two most important and also identifiable characteristics of vulnerable plaques [1–3]. Stary et al. published a series of papers and introduced the well-recognized American Heart Association (AHA) plaque classifications which served as the foundation for vulnerable plaque research [3]. Histopathological studies indicated that 65 µm could serve as a threshold value for vulnerable plaques [4–6]. It is fair to say that plaque cap thickness is the single most important factor people use to assess plaque vulnerability.

**Fig. 1:**
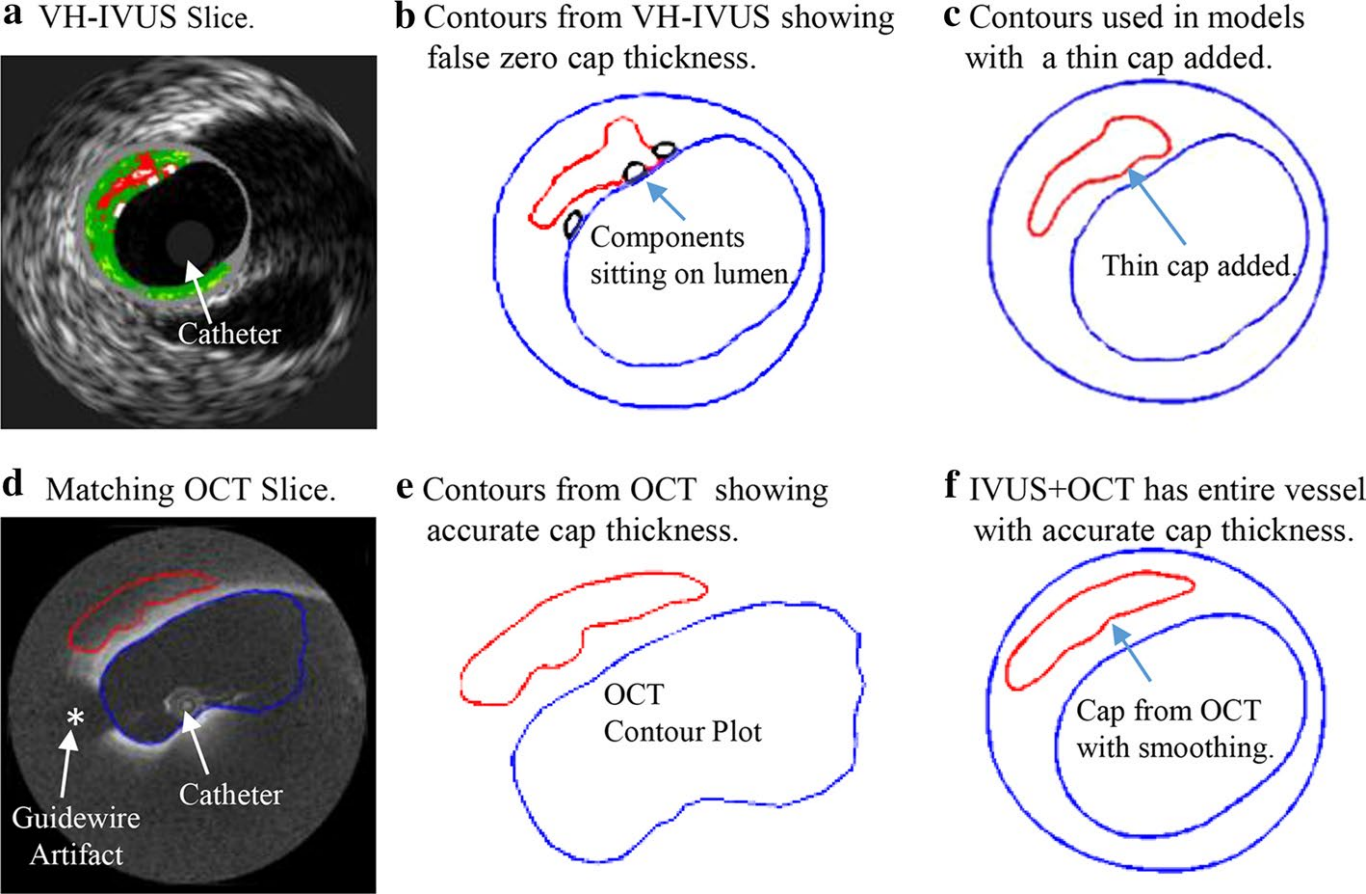
**a** Sample showing that IVUS gave inaccurate cap thickness and OCT provided accurate cap thickness. Calcification ignored. “*” indicates guidewire artifacts

Detecting vulnerable plaques in vivo and assessing their vulnerability have been great challenges for clinicians and the research community. Medical imaging plays an important role here. Coronary angiography is commonly used to detect the location and degree of stenosis of coronary arteries. However, angiography only “sees” blood. It could not see vessel structure and plaque components. Intravascular ultrasound (IVUS) is an invasive procedure and is used in selected patients who needs detailed confirmation of plaque shape, inflammation, etc., so that physicians can optimize treatment planning [7–9]. Gao et al. worked on the vessel border detection in intracoronary images (VBDI) using their novel privileged modality distillation framework of IVUS of lumen and media-adventitia borders and Optical Coherence Tomography (OCT) of lumen border. This privileged modality distillation converts the single-input-single-task learning problem from single-mode VBDI to a multiple-input-multiple-task problem and uses privileged image modality to help the learning model enter the target mode [10, 11]. The emergence of high-precision transducer, advanced dual-frequency catheter, and other devices will greatly contribute on integration IVUS and OCT. Zhang et al. proposed a high-frequency miniature ultrasonic transducer with high spatial resolution including the axial resolution of 36 µm and the lateral resolution of 141 µm [12, 13]. However, due to limited resolution of IVUS image (about 150–200 µm), it cannot provide accurate cap thickness quantifications (see Fig. 1). As an emerging imaging modality, OCT has a 15–20 µm resolution and can measure fibrous cap thickness more accurately and detect thin fibrous cap thickness < 65 μm, plaque rupture, and other rupture-prone features in patients [14, 15]. To check the feasibility of using OCT to measure the thickness of the fibrous cap within lipid-rich plaques, Kume et al. examined 35 lipid-rich plaques from 102 coronary arterial segments of 38 human cadavers. Cap thickness from OCT and corresponding histological images were compared and good correlation was (*y* = 0.97*x* + 28.49; *r* = 0.90; *p* < 0.001) [16]. Kini et al. investigated reproducibility of OCT fibrous cap thickness (FCT) measurements by independent observers. One hundred and seventy OCT pullbacks were analyzed by two independent observers and the intraclass correlation coefficient (ICC) of FCT was found to be 0.88 [95% confidence interval (CI) 0.80 to 0.93] [17]. In one clinical application and patient follow-up study, Hou et al. used IVUS and OCT to assess the effect of statin therapy on coronary plaque composition and plaque volume using serial multimodality imaging. OCT was used to assess FCT and IVUS was used to assess atheroma burden at 3 time points: baseline, at 6 months, and at 12 months. Thirty-six lipid-rich plaques in 27 patients with AT 60 mg and 30 lipid-rich plaques in 19 patients with AT 20 mg were enrolled in this study. AT 60 mg induced greater reduction in low-density lipoprotein cholesterol compared with AT 20 mg. OCT revealed continuous increase in FCT from baseline to 6 months and to 12 months in both groups. Plaque burden did not change significantly in both groups [18]. Jang et al. used OCT to measure cap thickness from three patient groups: Group A: recent acute myocardial infarction (AMI); Group B: acute coronary syndromes (ACS) constituting non–ST-segment elevation AMI and unstable angina; and Group C: stable angina pectoris (SAP). OCT imaging was performed with a 3.2F catheter. 57 patients (20 with AMI, 20 with ACS, and 17 with SAP) had analyzable images. The median value of the minimum thickness of the fibrous cap was 47.0, 53.8, and 102.6 µm, respectively (*p* < 0.034) [19]. These work clearly demonstrated the ability of OCT in identifying thin cap fibroatheroma (TCFA) and quantifying cap thickness with high accuracy.

In our previous modeling study using IVUS images, Fig. 1 gave an example, showing that IVUS gave inaccurate cap thickness and OCT provided high accuracy cap thickness quantifications [20]. That was the motivation of the current research: we would like to use real patient data to demonstrate differences between IVUS and OCT cap thickness measurements.

The shortcoming of OCT is that it cannot “see” the whole vessel due to its limited penetration (1–2 mm). Meanwhile, IVUS can “see” the whole vessel with limited resolution. Those two are often used together in the detection and identification of vulnerable plaques [21, 22]. By merging IVUS and OCT together, we can obtain whole vessel morphology from IVUS and superior resolution from OCT and provide better accuracy for fibrous cap quantifications. Guo et al. demonstrated the potential of using biomechanical models based on IVUS + OCT images which provided more accurate stress/strain calculations which could be used for better plaque assessment [20].

It is technically difficult to get both IVUS and OCT data since the data acquisition would require two separate catheterizations, one for IVUS and one for OCT. Currently, imaging device which could do both IVUS and OCT is not yet available in most hospitals. Both IVUS and OCT catheterization procedures are invasive and expansive. It is even more challenging to get follow-up data, since it is extremely difficult to get patients to come back to do two catheterizations just for research purposes. We were fortunate that Drs. Mintz and Maehara at The Cardiovascular Research Foundation (CRF), Columbia University provided us the IVUS and OCT data set from 10 patients (existing data, de-identified). Three hundred and forty-eight (348) high-quality slices with fibrous cap and lipid core were identified for analysis.

The aim of this study was to use OCT data as the benchmark to obtain patient-specific coronary plaque cap thickness data and evaluate the differences between OCT and IVUS fibrous cap quantifications. A cap index was also introduced as a quantitative measure of plaque vulnerability. IVUS and OCT cap index values were compared. Cap index values at baseline (called Time 1, or T1) and follow-up (called Time 2, or T2) were also investigated to observe the trend of plaque vulnerability changes.

## Results

### Cap thickness by IVUS has large difference from cap thickness by OCT

**Fig. 2:**
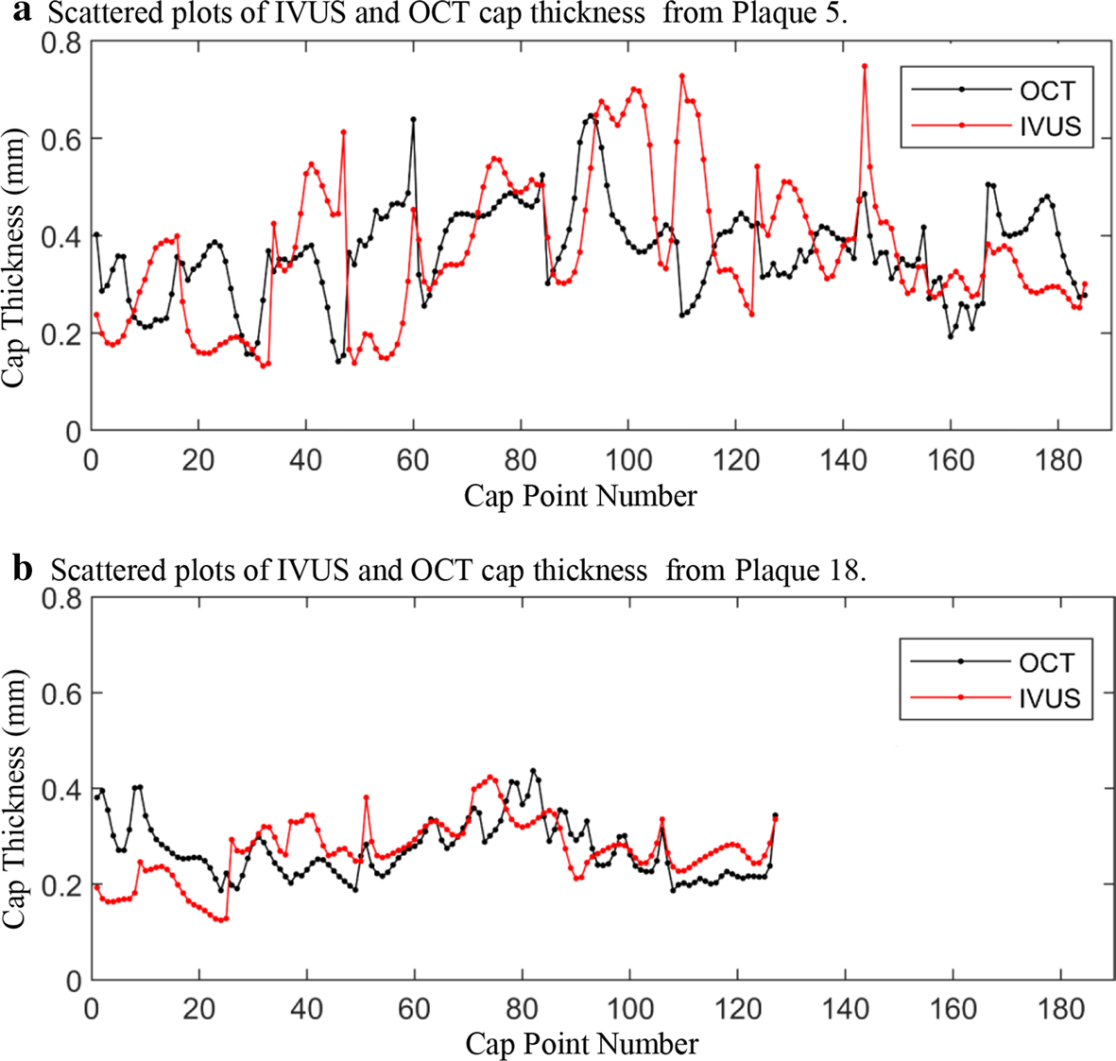
Scattered plots of cap thickness from two sample plaques showing point-to-point IVUS and OCT differences. Black dot: OCT; red dot: IVUS. Data points were connected by lines for better viewing

Patient morphological data and formulas for mean and minimum cap thickness (Min CapT) calculation and comparisons are provided in the Methods section. Table 1 summarizes mean and minimum cap thickness values for the 20 plaques using segmented IVUS and OCT contours. Each patient had baseline (T1) and follow-up (T2) data which were listed as two plaques in Table 1. The Kolmogorov–Smirnov tests were performed to check data normality. All data sets used in our analyses were normal and homogeneous. Results from paired t tests showed that the differences between OCT and IVUS cap thickness were statistically significant (*p* = 0.024 < 0.05 for Min CapT, *p* = 0.031 < 0.05 for Mean CapT). The average of OCT mean cap thickness of the 20 plaques was 0.382 mm and the average mean cap thickness from IVUS data was 0.375 mm. While the difference of the average values was a mere 1.83%, pointwise comparisons of IVUS and OCT cap thickness using Formula (3) indicated that the relative errors for the 20 plaques ranged from 21.37% to 73.91% (the average of was 35.76%). Figure 2 gave a point-by-point IVUS and OCT cap thickness plot for Plaque 5 and 18 showing the pointwise differences, while the IVUS and OCT cap thickness mean values were the same.

**Table 1:** Summary of mean and minimum cap thickness values for the 20 plaques

Plaque	Mean CapT			Min CapT		
	OCT	IVUS	Relative error (%)	OCT	IVUS	Relative error (%)
Plaque 1	0.298	0.343	21.37	0.128	0.193	50.78
Plaque 2	0.273	0.339	39.60	0.092	0.156	69.57
Plaque 3	0.385	0.398	33.75	0.200	0.183	8.50
Plaque 4	0.314	0.395	41.35	0.098	0.151	54.08
Plaque 5	0.368	0.368	33.03	0.141	0.132	6.38
Plaque 6	0.336	0.361	29.95	0.208	0.177	14.90
Plaque 7	0.526	0.442	38.00	0.268	0.134	50.00
Plaque 8	0.533	0.403	40.13	0.291	0.142	51.20
Plaque 9	0.298	0.401	40.82	0.159	0.247	55.35
Plaque 10	0.369	0.333	33.97	0.125	0.105	16.00
Plaque 11	0.343	0.236	44.47	0.177	0.041	76.84
Plaque 12	0.401	0.241	42.99	0.260	0.023	91.15
Plaque 13	0.603	0.497	31.28	0.324	0.141	56.48
Plaque 14	0.593	0.565	27.81	0.339	0.176	48.08
Plaque 15	0.300	0.348	37.71	0.122	0.025	79.51
Plaque 16	0.388	0.314	28.78	0.149	0.077	48.32
Plaque 17	0.283	0.237	25.26	0.127	0.124	2.36
Plaque 18	0.271	0.271	21.45	0.186	0.125	32.80
Plaque 19	0.380	0.358	29.50	0.228	0.136	40.35
Plaque 20	0.373	0.648	73.91	0.249	0.340	36.55
Max	0.603	0.648	73.91	0.339	0.340	91.15
Min	0.271	0.236	21.37	0.092	0.023	2.36
Ave. ± SD	0.382 ± 0.100	0.375 ± 0.102	35.76 ± 11.01	0.194 ± 0.073	0.141 ± 0.071	44.46 ± 24.50

Baseline plaque and follow-up plaque each counted as one plaque. Unit for cap thickness: mm

Minimum cap thickness for each plaque was defined as the minimum value of available cap points for that plaque with all slices included. The average of OCT minimum cap thickness of the 20 plaques was 0.194 mm and that average for IVUS data was 0.141 mm. The difference of the two average values was 27.32%. Plaque-wise comparisons of IVUS and OCT minimum cap thickness indicated that the relative errors for the 20 plaques ranged from 2.36% to 91.15% (the average of was 44.46%). Pointwise comparison for minimum cap thickness does not apply, since plaque minimum cap thickness was defined as the minimum of all cap points of the given plaque.

### Vulnerability assessment using cap index and IVUS/OCT cap index comparisons

**Fig. 3:**
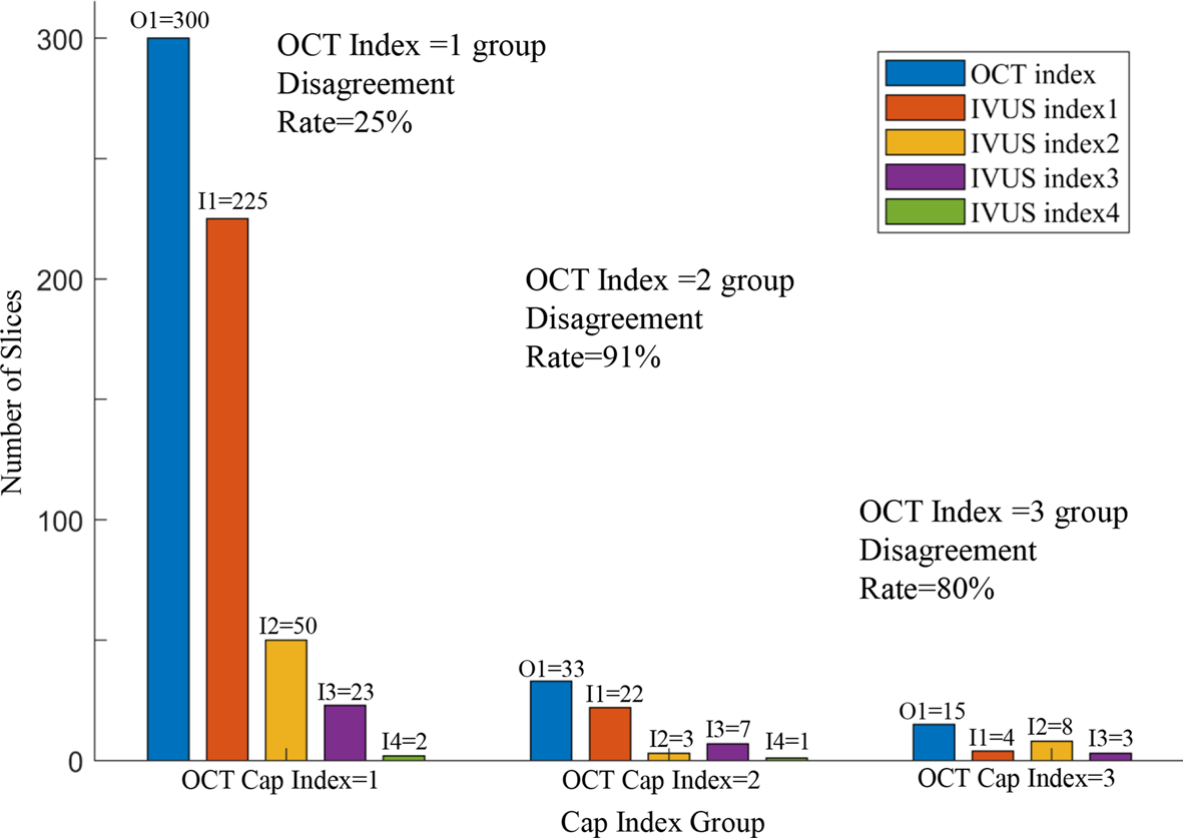
Bar plots of 3 OCT cap index groups and their corresponding IVUS cap index distributions. Disagreement rates were given for each group. O1 = OCT Cap Index = 1; I1 = IVUS Cap Index = 1; O2 = OCT Cap Index = 2; I2 = IVUS Cap Index = 2. Other notations were defined similarly

Cap index introduction, definition, and index value assignments are provided in the Methods section. Table 2 gives cap index assignment results using OCT data for all 348 slices and their corresponding IVUS cap index assignments. Figure 3 gives a bar plot for the three OCT index groups with their corresponding IVUS cap index distributions for each group. The disagreement rate is the percentage of the number of slices whose IVUS indices differed from their OCT indices for that group. For all 348 slices, the numbers of slices with OCT cap index = 1, 2, 3, 4 were 300, 33, 15, and 0, respectively. For each group with the same OCT cap index value, the numbers of slices with the 4 IVUS cap index values were given. For the OCT cap index = 1 group, the numbers of slices with IVUS cap index = 1, 2, 3, 4 were 225, 50, 23, and 2, respectively. The disagreement between OCT and IVUS assignments was 25%. For OCT cap index = 2 and 3 groups, the disagreement rates between OCT and IVUS assignments were as high as 91% and 80%. While the overall disagreement rate for all slices was 33.6%, the disagreement rate for OCT cap index = 2 and 3 groups (slightly unstable and moderately unstable) was 87.5%. For the 348 slices, 23.9% slices had IVUS cap index values overestimated OCT index values. The underestimation rate was 9.8%. For the OCT cap index = 2 and 3 groups (total 48 slices), IVUS cap index overestimated plaque vulnerability by 16.7%. The underestimation rate was 70.8%. The precision, recall, specificity, and negative predictive value (NPV) values for fibrous cap detection using IVUS against OCT are given in Table 3.

**Table 2:** Number of slices for each OCT cap index group and corresponding IVUS cap index values

OCT cap index	Number of slices						
	OCT slices	IVUS index = 1	IVUS index = 2	IVUS Index = 3	IVUS index = 4	Slices with index changed	Change rate (%)
1	300	225	50	23	2	75	25.00
2	33	22	3	7	1	30	90.91
3	15	4	8	3	0	12	80.00
4	0	–	–	–	–	–	–
Total	348	251	61	33	3	117	33.62

**Table 3:** Prediction parameter values of IVUS detecting cap index based on OCT criteria

	Index 1(%)	Index 2(%)	Index 3(%)	Index 4
Precision	89.64	4.92	9.09	None
Recall	75.00	9.09	20.00	None
Specificity	45.83	81.59	90.99	None
NPV	22.68	89.55	96.19	None

### Vulnerability assessment from baseline (T1) to follow-up (T2)

Table 4 lists the cap index assignments for each patient at *T*1 and *T*2 using OCT and IVUS data, respectively. The index assignment was made using the minimum cap thickness for every plaque, i.e., the minimum of all slice minimum cap thickness of the plaque. The index change (Δ_Index_) from *T*1 to *T*2 for every patient was obtained. It is of clinical significance to know if a plaque was getting more vulnerable (Δ_Index_ > 0), more stable (Δ_Index_ < 0) or unchanged (Δ_Index_ = 0). Based on OCT Δ_Index_ data, 2 plaques became more vulnerable; 5 plaques had the same cap index; and 3 plaques became more stable. Comparing IVUS Δ_Index_ with OCT Δ_Index_, the agreement rate was shown by “Trend Comparison” and it was only 20%. In another word, for the 10 patients studied, IVUS data gave wrong vulnerability change assessment for 8 patients.

**Table 4:** Plaque cap index assignments using minimum cap thickness for the plaque at baseline (T1) and follow-up (T2)

Patient	OCT			IVUS			IVUS and OCT Δ_index_ Equal?
	T1 Index	T2 Index	Δ_Index_	T1 Index	T2 Index	Δ_Index_	
P1	3	3	0	2	2	0	Y
P2	2	3	1	2	2	0	N
P3	3	1	− 2	3	2	− 1	N
P4	1	1	0	3	3	0	Y
P5	2	3	1	3	2	− 1	N
P6	2	1	− 1	4	4	0	N
P7	1	1	0	3	2	− 1	N
P8	3	3	0	4	3	− 1	N
P9	3	2	− 1	3	3	0	N
P10	1	1	0	3	1	− 2	N

Δ_index_ = (Plaque Cap Index at T2)–(Plaque Cap Index at T1)

## Discussion

### Significance of quantifying cap thickness and comparison with current literature

Detection, identification, and assessment of vulnerable plaques are of the utmost importance for cardiovascular research and public health. Cap thickness is one of the very few morphological characteristics linked to plaque vulnerability measurable in vivo. IVUS is the most common imaging modality currently used in clinical practice. Due to the limitation of IVUS resolution, IVUS is not able to detect TCFA with desired accuracy and reliability. Plaque assessment and diagnosis are most done based on clinician’s experiences. As OCT is getting acceptance in clinical use, efforts of combining IVUS and OCT have been made by several groups to identify vulnerable plaques and quantify thin cap thickness. Recent reviews of using IVUS and OCT to detect vulnerable plaque features can be found from Fujii et al. [23] and MacNill et al. [24].

Papers in the current literature often focused on clinical diagnosis and identification of TCFA, less on getting plaque morphology and fibrous cap outlines (contours) which are important for modeling and further biomechanical investigations [25, 26]. In the paper by Kume et al. 35 lipid-rich plaques from 102 coronary arterial segments of 38 human cadavers were examined to check the feasibility of using OCT to measure the thickness of the fibrous cap within lipid-rich plaques, and good correlation for cap thickness from OCT and histology was found. The thickness of the fibrous cap was measured at the thinnest part of each plaque [16]. No cap contour (see our Fig. 1) was drawn. Kini et al. investigated reproducibility of OCT FCT measurements by independent observers. The paper actually focused on plaque classifications (lipid-rich, TCFA, or fibrocalcific) by 2 observers and determined that ICC of FCT was found to be 0.88 [95% confidence interval (CI) 0.80 to 0.93] [17]. Minimum cap thickness was used in their study, as well. The paper by Hou et al. was a follow-up study and the use of IVUS and OCT was close to what we did in our paper: OCT was used to quantify cap thickness, and IVUS was used to quantify plaque burden. OCT was able to detect change in cap thickness in their follow-up which is not possible for IVUS [18]. The purpose of Jang et al.’s study was clinical: measure cap thickness from three patient groups to find if there are cap thickness differences among the three groups: Group A: AMI; Group B: ACS constituting non-ST-segment elevation AMI and unstable angina; and Group C: SAP [19]. Ex vivo comparison for plaque classification found that OCT had a higher diagnostic sensitivity and accuracy to identification of TCFA marginally exceeding IVUS [27].

Our work is distinctive in several aspects: (a) we obtained clear cap and whole plaque morphology outlines which will serve as biomechanical modeling basis for further investigations, while none of the other studies performed the same task; (b) we provided clear comparison of IVUS and OCT cap thickness quantifications which also cannot be found in the current literature. The comparison will help clinicians and researchers to know the errors associated with estimations using IVUS data. This will also serve as justification for further acceptance of OCT in clinical practice.

It was not a surprise to find that there are statistically significant differences between IVUS and OCT data. While the mean value difference was only 1.83%, point-to-point comparison indicated that the IVUS and OCT cap thickness difference was 35.76%. Figure 2 gave scattered plots of all cap points for 2 plaques to demonstrate the point-to-point differences.

Since plaque rupture happens normally at the weakest location of the artery, it makes sense to pay special attention to the minimum cap thickness for each plaque. While the mean of the minimum cap thickness of the 20 plaques from IVUS and OCT differed by 27.32%, the mean of the patient-specific IVUS vs. OCT differences was 44.46%, ranging from 2.36% to 91.15%. This result indicated that one should be more cautious when assessing minimum cap thickness using IVUS.

### Significance of accurate cap thickness data for plaque vulnerability assessment

One objective of research is to generate results that are easy to understand and easy to use. Cap index was introduced for this purpose. While IVUS cap index values differed from OCT for the stable group only by 25% (the OCT index = 1 group, *n* = 300), the difference was 91% for the slightly unstable group (OCT cap index = 2, *n* = 33) and 80% for the moderately unstable group (OCT cap index = 3, *n* = 15). Assessment for more vulnerable plaques is more relevant in clinical practice for diagnosis and treatment decisions. In previous studies, the accuracy and feasibility of IVUS and other IVUS related techniques for identifying OCT-derived TCFA has been fully demonstrated. Miyamoto et al. worded on 81 coronary lesions with plaque burden > 40% with both IB-IVUS and OCT. 49% TCFA was identified by OCT and plaque component was used to analyzed the correlation of TCFA and non-TCFA using IB-IVUS [21]. The correlation and diagnostic accuracy between IVUS and OCT recognition fibrous caps was studied by Kubo et al. They used VH-IVUS and OCT images in 96 target lesions and found that the sensitivity and specificity of VH-IVUS to identify TCFA as determined by OCT were 89% and 86% [28]. On this basis, the differences between IVUS and OCT under the fibrous cap index were highlighted, and the differences in vulnerability assessment and prediction of IVUS and OCT were compared, providing a basis for the biomechanical analysis of plaque.

### Application of follow-up cap thickness data for plaque vulnerability trend assessment

Another important goal in vulnerable plaque management and research is to identify the trend of vulnerability changes with which medication and treatment strategies could be modified properly for better health of the patients. Diletti et al. studied coronary atherosclerosis progression and regression using combined IVUS and OCT (24 patients, 27 lipid-rich plaques at baseline, 6-month follow-up) [29]. At 6-month follow-up, 22 (81%) did not show any significant change. There were no significant changes in percent NC and fibrous cap thickness in the 3 bifurcation regions between baseline and follow-up examinations. While values for 14 geometrical and compositional parameters were listed, cap thickness data were not given in the paper. The study by Xie et al. presented results which are closest to our findings [30]. They compared OCT and IVUS for evaluation of coronary plaque progression and regression (88 lipid-rich plaques from 65 patients, 12-month follow-up). Fibrous cap thickness on OCT was negatively correlated with total atheroma volume on IVUS (*r* = − 0.28, *p* = 0.009), but not with percent atheroma volume (*p* = 0.84). OCT and IVUS measurements of cap thickness, maximum and mean lipid core arc, lipid core length, and lipid index at both baseline and follow-up were reported. There were no significant correlations between the changes in OCT measures and the changes in IVUS results. That is consistent with our findings.

Our unique patient follow-up data set allowed study on cap index assignments for both *T*1 and *T*2 to identify the trend. Similar results using IVUS and OCT combined data are not available in the current literature. Results in Table 5 showed that IVUS trend assessment differed from OCT by 80%. While our data set is small (348 slices from 10 patients), this is at least enough for us to keep in mind that IVUS has limitations in plaque vulnerability trend assessment.

**Table 5:** Patient demographic and clinical information

Patient ID	Age	Sex	BP (mmHg)	Vessel segment	Diagnosis history	Follow-up days
P1	80	F	71–138	RCA	HT DM	304
P2	70	M	84–155	RCA	HT	273
P3	65	F	63–149	RCA	DM	220
P4	66	M	89–150	LCX	DM	290
P5	81	M	69–112	LAD	HT	182
P6	73	M	55–150	LCX	HT HL	248
P7	74	F	62–151	LAD	HT DM HL	244
P8	62	F	79–117	LAD	HL	195
P9	61	M	78–128	LCX	HT DM HL	283
P10	72	M	80–143	LCX	HT DM HL	272

*M* male, *F* female, *HT* hypertension, *DM* diabetes mellitus, *HL* hyperlipidemia

### Potential clinical applications

The purpose of this paper was to provide some accurate real patient coronary plaque cap thickness data (small size) by OCT and their comparison with that from IVUS. The clinical significance is self-clear, since cap thickness is the most-watched and most-desired data for vulnerable plaque research. However, our unique approach could provide plaque morphology contours based on combined IVUS/OCT data for model construction use. For research purpose, accurate cap thickness could improve plaque stress/strain calculations from computational models which can be used to gain better understanding of mechanisms governing plaque progression and rupture. The final goal is to predict plaque progression and rupture and prevent tragic cardiovascular events before they actually happen. The direct clinical use of our contours is limited. There is still a long way to go for our modeling research results which could be implemented for actual clinical applications.

With its high resolution and ability to detect TCFA and quantify thin cap, OCT has great potential in diagnosis and treatment decision-making process. Detecting vulnerable plaque is now possible with OCT. We can also monitor plaque cap thickness development and adjust treatment plan as needed [18]. For clinical applications, the question is actually not that people could have a choice of using either IVUS or OCT. It is what is actually available for the physicians to use in hospitals. Currently, IVUS is available in most hospitals and widely used as a major diagnosis tool for physicians to exam patients and make treatment decisions (stenting or taking medication). At the same time, OCT is slowly moving closer to be accepted and become available for use in hospitals. However, it is still relatively new and not as readily available. Equipment which can perform both IVUS and OCT simultaneously is being developed and could become available in the near future.

## Limitations

The present work has several limitations. First, we examined 348 slices with lipid core and fibrous cap from 10 patients with baseline and follow-up data. Large-scale patient studies are needed to have statistical significance at patient level. Second, although expert-based and convolutional neural networks (CNN)-based machine learning methods were used for image processing, lack of histological data to serve as gold standard is a limitation. Larger OCT data set is also needed to improve the accuracy and reliability of CNN-based methods. Extensive man power needed for the expert-based manual segmentation to generate the gold standard for machine learning methods is also a limitation. The clinical significance of our results is limited by the small size of patients. Large-scale patient studies are needed for further improvement and validation.

## Conclusions

Our preliminary results demonstrated that there were significant differences between IVUS and OCT plaque cap thickness measurements. Measurements from IVUS should be taken with precaution and confirmed by OCT when possible. Accurate cap thickness could be used to generate accurate plaque stress/strain data and predict plaque growth and vulnerability. Large-scale patient studies are needed to confirm our findings.

## Methods

### Image data acquisition, co-registrations, and segmentation

Data from patients with coronary heart disease who underwent IVUS, OCT, and angiography (from April 2017 to November 2018) in their 3 epicardial coronary vessels were collected at CRF using protocol approved by the local institute and informed consents were obtained from the patients. CRF has been conducting coronary patient follow-up studies for many years. From their data pool, CRF has identified 10 patients at baseline and 8-month (average 251 days) follow-up. IVUS and OCT imaging were performed the same day. Patient demographic data are shown in Table 5. Selected patients were with stable angina pectoris undergoing percutaneous coronary intervention (PCI). Patients with acute coronary syndrome, severe calcified lesion, chronic total occlusion, or chronic kidney disease (Cr > 1.5 mg/dl) were excluded. IVUS examination was performed after 0.2 mg of intracoronary nitroglycerin. The IVUS catheter was advanced as far as possible using a commercially available IVUS system: a 40 MHz IVUS catheter (OptiCross, Boston Scientific Corporation, Natick, Massachusetts) with motorized pullback at 0.5 mm/s. OCT images were acquired with ILUMIEN OPTIS System, and Dragonfly or Dragonfly JP Imaging Catheter (St. Jude Medical, Westford, Massachusetts).

**Fig. 4:**
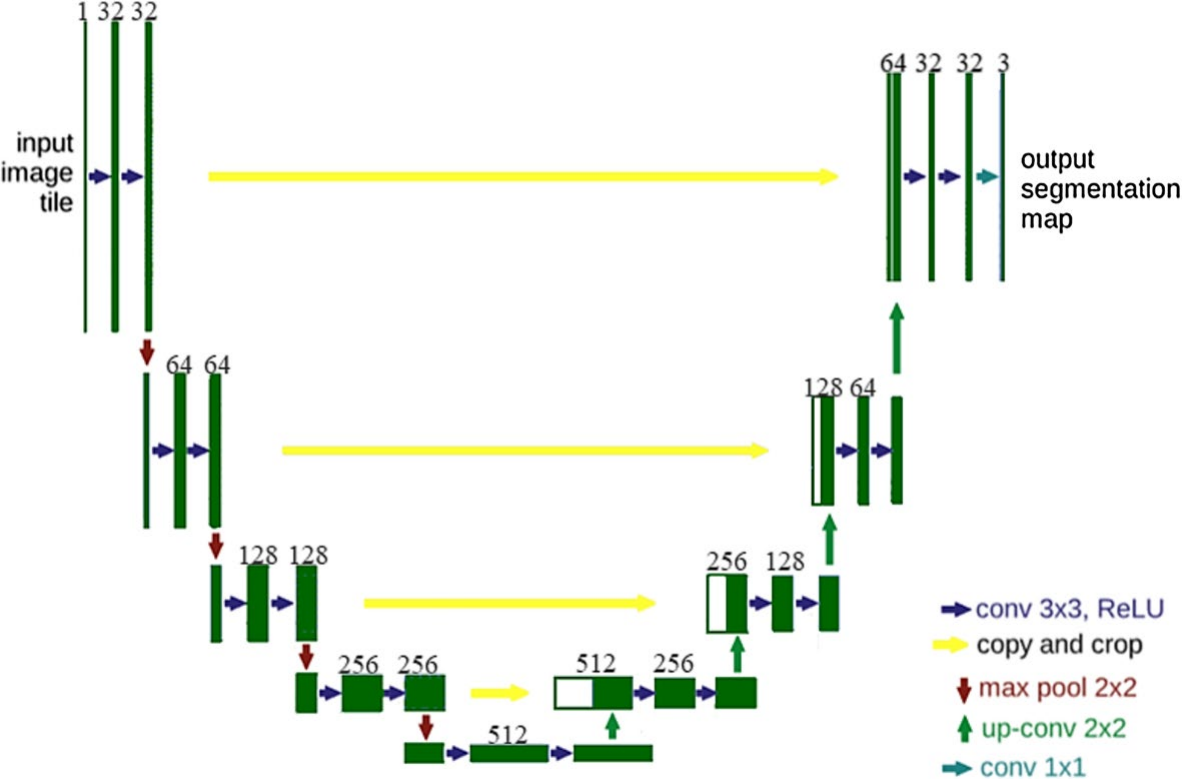
An illustration diagram of the U-Net architecture. Each dark green box corresponds to a multi-channel feature map and the number of channels is noted on top of the box. White boxes represent copied feature maps. The arrows denote work flow of different operations

Manual segmentations were performed by two experts which provided gold standard for CNN segmentation method. Each image slice was segmented into three plaque components: fibrotic region, necrotic core (lipid), and dense calcium (calcification). CNN-based segmentation of OCT images were performed following published procedures [31, 32]. The CNN algorithm was based on the U-Net architecture, and it uses convolution, pooling, and fully connected layers to extract the features of images of different scales, which has achieved remarkable results in medical image processing, computer vision, and other fields [33, 34]. In OCT image, lipid and fibrous components of the plaques are unevenly distributed. CNN method can extract the details of low-level information, effectively deal with class imbalance problems using Batch Normalization and Adagrad, realize the encapsulation of feature extraction, achieve better classification effect, and ensure the accuracy of classification. Figure 4 gives a diagram for U-Net architecture used in this paper. U-Net architecture is named for its U-shaped structure composed of compression path and storage path, which can be used to achieve image semantic segmentation. Parameters used in U-Net segmentation process are as follows: the contracting path included the repeated application of two 3 × 3 convolutions, each followed by scaled exponential linear units (SeLU) and a 2 × 2 max pooling operation with stride 2 for downsampling. In each downsampling step, the number of feature channels was doubled. Each step in the expansive path consisted of an upsampling of the feature map followed by a 2 × 2 convolution a concatenation with the correspondingly cropped feature map from the contracting path, and two 3 × 3 convolutions, each followed by a SeLU. And cropping was applied. Finally, a 1 × 1 convolution was used to map each feature vector to three classes. Computer platform was Pytorch (version1.1.0) based on python 3.6 and cuda 9.0. Training time was 9–10 min for 1 epoch, 15–17 h for total. Testing time for 1 image was 1–2 s. Batch size was 2. Learning rate was 0.0002. Optimizer was Adagrad. We also used Batch Normalization to solve the gradient disappearance problems. Contour acquisition used the RGB three-way channel, getting the connected region and using eight-neighborhood tracking algorithm to get the boundary of the connected region. The errors for minimum and mean cap thickness between the gold standard and CNN-based segment method were 4.31% and 6.15%, respectively. The overall U-Net average classification accuracies for fibrous tissue was 94.85%. The average prediction accuracy for lipid was 84.38%. The overall average accuracy differentiating lipid and fibrous tissue was 93.63%. The average errors for cap thickness and minimum cap thickness quantifications were 7.54% and 12.48, respectively. OCT and IVUS data were analyzed by two independent observers and the ICC of cap thickness were found to be 0.84 [95% confidence interval (CI) 0.76 to 0.90] for OCT and 0.76 (95% CI 0.63 to 0.85) for IVUS, respectively. Those were consistent with the current literature [17, 31]. Human errors could be a contributing factor to the disagreements between OCT and IVUS measurements.

**Fig. 5:**
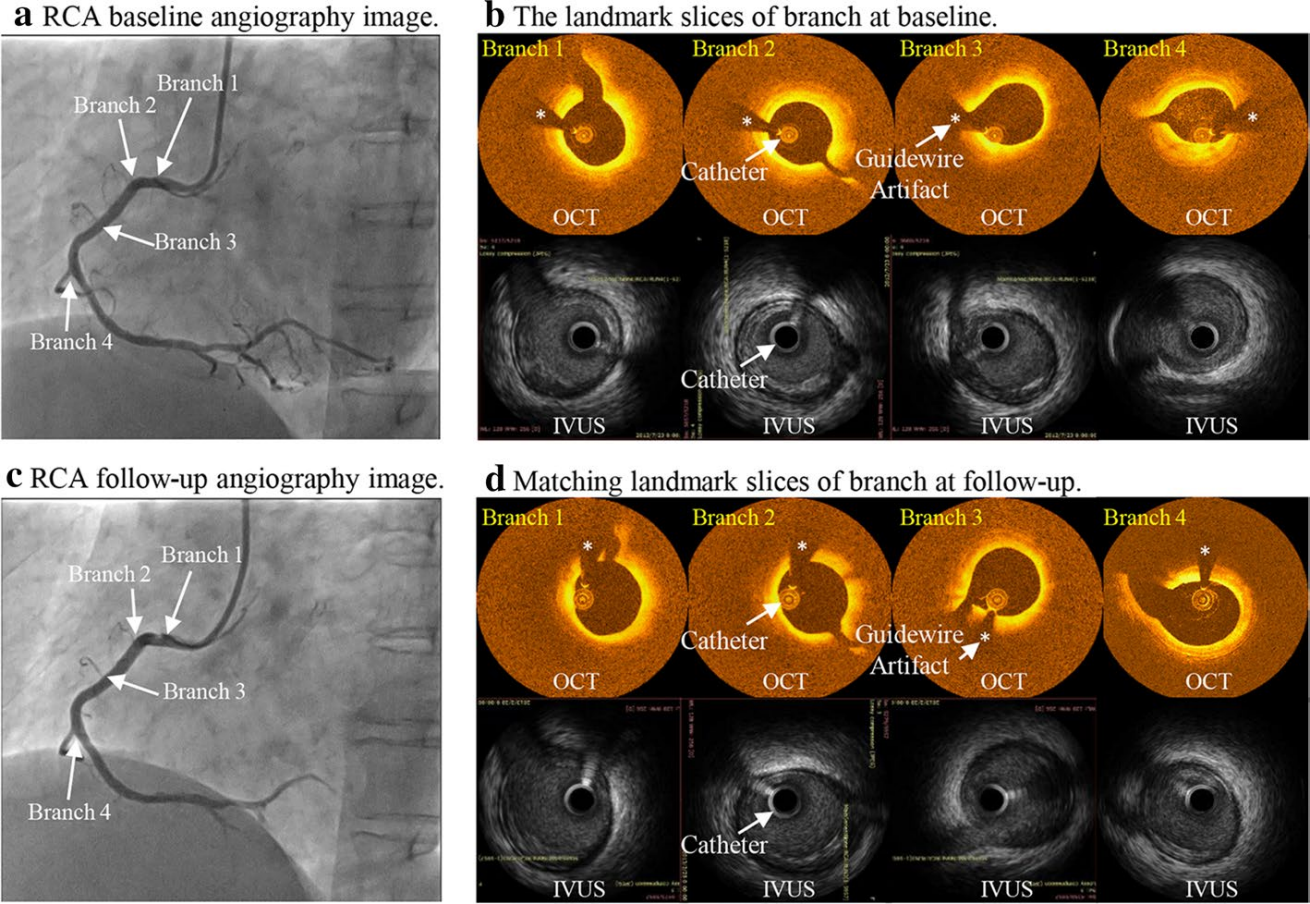
Co-registration of IVUS and OCT images with angiography at baseline and follow-up. Catheter was marked. “*” indicates guidewire artifacts

**Fig. 6:**
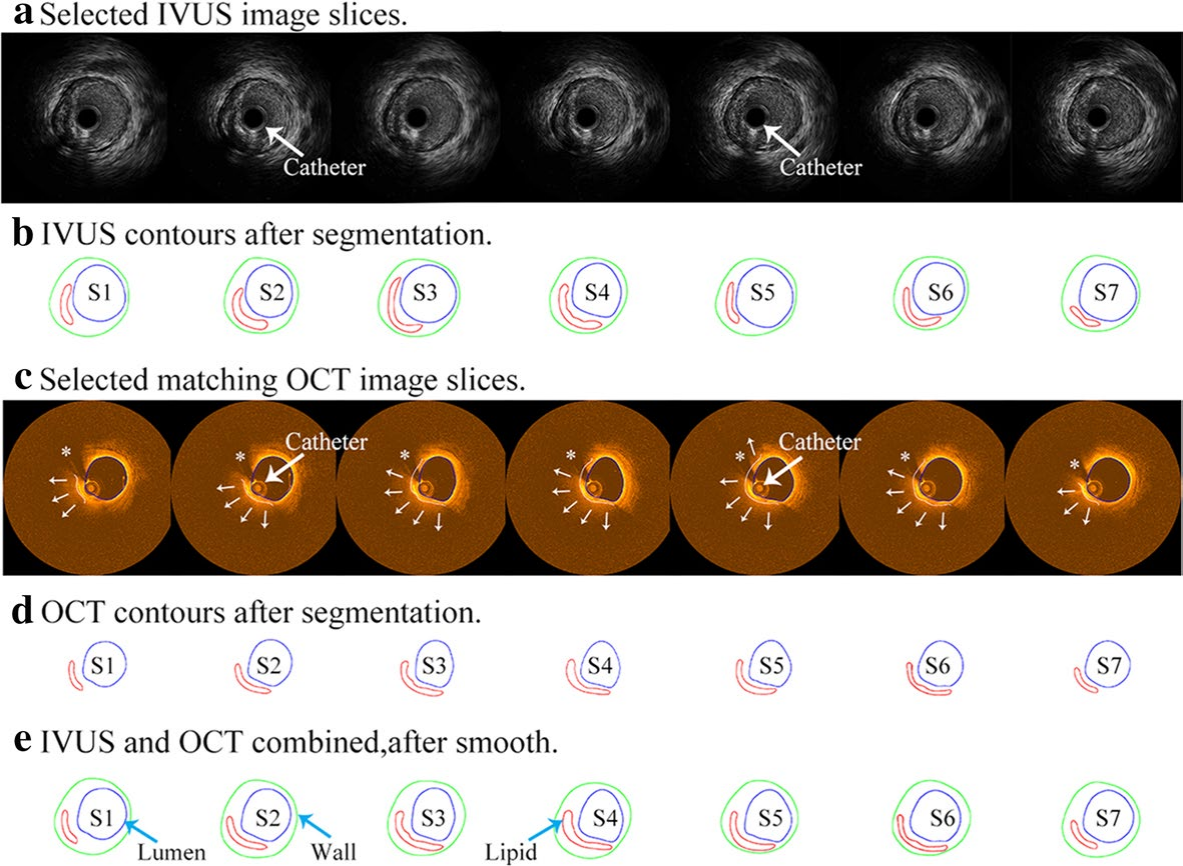
IVUS and OCT segmentation and merging process. **a**: Selected IVUS image slices; **b**: IVUS contours after segmentation; **c**: Selected matching OCT image slices, guidewire artifacts (*), lipid region (marked by small white arrows), lipid edge (white curve), and lumen (blue curve); **d**: OCT contours after segmentation; **e**: IVUS and OCT combined, after smooth. Blue: lumen; Red: lipid; Green: outer wall. Catheter was marked by large white arrows in the figure

Co-registration of IVUS and OCT slices was performed using fiduciary points such as side branches, bifurcations, and calcifications. Matched IVUS and OCT slices were combined together to form IVUS and OCT slices with IVUS providing whole vessel morphology and OCT providing accurate cap thickness quantifications. Figure 5 gives an example showing the co-registration IVUS and OCT images with angiography and their co-registration between T1 and T2. Figure 6 shows sample slices from a patient to demonstrate IVUS and OCT segmentation and merging process. Plots given by Fig. 6e demonstrate several sample IVUS and OCT slices whose lumen and component contours were taken from OCT, while wall was taken from IVUS.

### Determination of cap thickness and difference between IVUS and OCT data

**Fig. 7:**
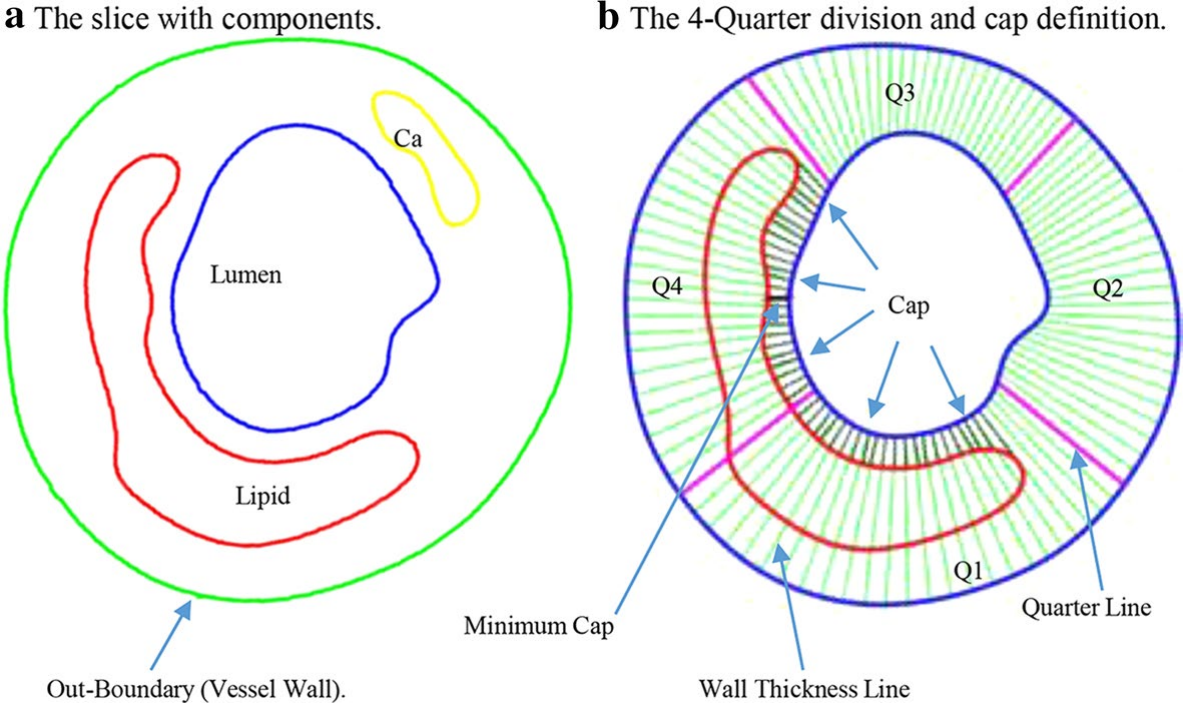
The schematic plot showing the four-quarter method to define cap and wall thickness. Quarter 1 (Q1) was chosen as the side towards the myocardium

Three hundred and forty-eight (348) matched slices with fibrous cap and lipid core(s) were selected for data analysis and comparison. Due to irregular plaque morphologies, after the contours was separated, a four-quarter even-spacing method was introduced to quantify vessel wall thickness and cap thickness with data points distributed on the lumen and slice out-boundary more evenly (See Fig. 7). First of all, location of myocardium was determined by CRF experts from viewing IVUS/OCT and angiography movies. The location of myocardium was marked on OCT/IVUS slices. For every slice, the lumen was divided into 100 lm points evenly. The lumen was divided into 4 quarters, with Quarter 1 (Q1) as the side towards the myocardium. Each quarter contained 25 points, with the quarter dividing points, as shown in Fig. 7. The out-boundary contour (also called vessel wall) was divided into 4 quarters. The shortest distance method was used to locate the 4 wall dividing points, so that they had the shortest distance to their matching lumen dividing points. After that, each quarter of the wall contour was divided evenly into 25 parts matching lumen divisions. Wall thickness was defined as the distance between the lumen point and its corresponding out-boundary point. Cap thickness (CapT) was the part of the connecting line from lumen contour to the lipid edge near the lumen. Only those points covering a lipid core were called cap points with cap thickness values assigned and recorded for analysis.

Point-to-point differences (in absolute value) between OCT and IVUS cap thickness were calculated for every matched data point *x*_*i*_:

1 $$ {\text{Cap}}\;{\text{ thickness}}\;{\text{ difference}}\;{\text{ at}}\;{\text{ data}}\,{\text{ point}}\;x_{i} \; = \;\Delta_{cap} \left( {x_{i} } \right)\; \, = \;\left| {{\text{CapT}}\__{{{\text{OCT}}}} \left( {x_{i} } \right) \, - {\text{CapT}}\__{{{\text{IVUS}}}} \left( {x_{i} } \right)} \right|. $$

The mean values of the point-to-point differences for every slice, every plaque, and all the patients were calculated by:

2 $$ {\text{Slice}}\;{\text{Mean}}\;{\text{Cap}}\;{\text{Thickness}}\;{\text{Difference}}\; = \;\sum\nolimits_{i\; = \;1}^{n} {\Delta_{{{\text{cap}}}} \;(x_{i} )/n,} $$

3 $$ {\text{Plaque}}\;{\text{Mean}}\;\Delta_{cap} \;(x_{i,j} )\; = \;\left( {\sum\nolimits_{j\; = \;1}^{m} {\sum\nolimits_{i\; = \;1}^{n} {\Delta_{{{\text{cap}}}} \;\left( {x_{i,j} } \right)} } } \right)/\sum\nolimits_{j\; = \;1}^{m} {n_{j} } , $$

4 $$ {\text{All}}\;{\text{Plaque}}\;{\text{Mean}}\;\Delta_{cap} \;(x_{i,\;j,\;k} )\; = \;\sum\nolimits_{k = 1}^{L} {\sum\nolimits_{j\; = \;1}^{{m_{k} }} {\sum\nolimits_{i\; = \;1}^{{n_{j} }} {\Delta_{cap} \;(x_{i,\;j,\;k} )} } /\sum\nolimits_{k\; = \;1}^{L} {\sum\nolimits_{j\; = \;1}^{{m_{k} }} {n_{j,k} ,} } } $$

where ${x}_{i}$, ${x}_{i,j}, {x}_{i,j,k}$ stand for every matched data point at slice, plaque, and all plaque levels; $i$,$j$,and $k$ are indices for point, slice and plaque; $n_{j} \;{\text{and}}\;(n_{{j,k}} )$ is the number of points on the $j$ th slice, $k$ th plaque; ${m}_{k}$ is the number of slices for the $k$ th plaque; $L$ is the number of plaques. It should be noted that cap thickness difference should always be calculated for every data point, and then sum up for all data points used. Mean Cap Thickness was also denoted as Mean CapT. Using OCT data as the gold standard, the relative error by IVUS measurement is given by:

5 $$ {\text{Relative Error}}\, = \,\left( {{\text{Mean CapT Difference between IVUS }}\& {\text{ OCT}}} \right) \, /{\text{ Mean CapT}}\__{{{\text{OCT}}}} . $$

Min CapT for a slice or a patient was defined as the minimum value of available cap points for that slice or patient, since one could assume that rupture would happen at the thinnest cap point.

### Cap index assignments and measure of plaque vulnerability

AHA has given qualitative plaque classifications which have widely accepted. However, well-accepted quantitative plaque vulnerability definitions are still to be established. In practice, cap thickness is the most-watched and used measurable feature in research and clinical diagnosis, especially with OCT getting acceptance [35]. Table 6 indicates the assignment method for cap index as a measure for plaque vulnerability and its comparison with AHA classifications. For each slice, cap index was assigned using minimum IVUS and OCT cap thickness, respectively. The differences between IVUS and OCT cap indices were compared.

**Table 6:** Cap index definition using plaque cap thickness related to plaque vulnerability and AHA plaque classifications

Cap index	Description	AHA type	Description	Vulnerability level
0	No component	Type I or Type II	Normal or slight intimal thickening	Very stable
1	Minimum cap thickness > 200 µm	Type III	Moderate intimal thickening, no extracellular lipid, calcification or significant inflammation	Stable
2	150 µm < minimum cap thickness ≤ 200 µm	Type IV, Vb and Vc	Small lipid core (< 30% of plaque size); calcification may be present; thick fibrous cap(> 150 μm);	Slightly unstable
3	65-m < min cap thickness ≤ 150 µm	Type Va	Moderate lipid core(30–40% of plaque size) and fibrous cap(65–200 μm); moderate intraplaque hemorrhage and inflammation	Moderately unstable
4	Min cap thickness ≤ 65 µm	Type VI	Large lipid core(> 40%); thin fibrous cap(< 65 μm); large intraplaque hemorrhage; extensive inflammation; previous plaque rupture	Highly unstable

## Statistical analysis

OCT and IVUS mean cap thickness and minimum cap thickness were compared to obtain the differences between OCT and IVUS data and the errors by IVUS data with its inherited resolution limitations. OCT data were used as the base when computing relative errors by IVUS. The Kolmogorov–Smirnov tests were performed to check data normality. Analysis of variance and paired* t* tests were used to check if the differences between IVUS and OCT data sets were statistically significant. A *p* value < 0.05 was considered statistically significant.

## Data Availability

The data used and analyzed during the current study are available from the corresponding author on reasonable request.

## Abbreviations

ACS: Acute coronary syndromes
AHA: American heart association
AMI: Acute myocardial infarction
CapT: Cap thickness
CNN: Convolutional neural network
CRF: Cardiovascular research foundation
DM: Diabetes mellitus
F: Female
FCT: Fibrous cap thickness
HL: Hyperlipidemia
HT: Hypertension
ICC: Intraclass correlation coefficient
IVUS: Intravascular ultrasound
M: Male
Min CapT: Minimum cap thickness
NPV: Negative predictive value
OCT: Optical coherence tomography
PCI: Percutaneous coronary intervention
Q1: Quarter 1
SAP: Stable angina pectoris
T1: Time 1
T2: Time 2
TCFA: Thin-cap fibroatheroma
VBDI: Vessel border detection in intracoronary images
